# Investigating the Forming Characteristics of 316 Stainless Steel Fabricated through Cold Metal Transfer (CMT) Wire and Arc Additive Manufacturing

**DOI:** 10.3390/ma17102184

**Published:** 2024-05-07

**Authors:** Yi Feng, Ding Fan

**Affiliations:** 1 Materials Science and Engineering College, Lanzhou University of Technology, Lanzhou 730050, China; yfenglut@foxmail.com; 2 Lanzhou Institute of Technology, Material Engineering College, Lanzhou 730050, China; 3 State Key Laboratory of Advanced Processing and Recycling of Non-Ferrous Metal, Lanzhou University of Technology, Lanzhou 730050, China

**Keywords:** formation characteristics, microstructure evolution, CMT, wire and arc additive manufacturing

## Abstract

Wire and arc additive manufacturing (WAAM), recognized for its capability to fabricate large-scale, complex parts, stands out due to its significant deposition rates and cost-effectiveness, positioning it as a forward-looking manufacturing method. In this research, we employed two welding currents to produce samples of 316 austenitic stainless steel utilizing the Cold Metal Transfer wire arc additive manufacturing process (CMT-WAAM). This study initially evaluated the maximum allowable arc travel speed (MAWFS) and the formation characteristics of the deposition bead, considering deposition currents that vary between 100 A and175 A in both CMT and CMT pulse(CMT+P) modes. Thereafter, the effect of the CMT+P mode arc on the microstructure evolution was analyzed using the EBSD technique. The findings indicate that the arc travel speed and deposition current significantly affect the deposition bead’s dimensions. Specifically, an increase in travel speed or a reduction in current results in reduced bead width and height. Moreover, the employment of the CMT+P arc mode led to a reduction in the average grain size in the mid-section of the sample fabricated by CMT arc and wire additive manufacturing, from 13.426 μm to 9.429 μm. Therefore, the components of 316 stainless steel produced through the CMT+P-WAAM method are considered fit for industrial applications.

## 1. Introduction

Additive manufacturing (AM) refers to a technology that shapes parts directly from a CAD model, setting it apart from traditional manufacturing methods such as turning and milling. AM is celebrated for its streamlined processes, enhanced material efficiency, and increased design flexibility. Specifically, wire and arc additive manufacturing (WAAM) employs an arc to melt wire, emerging as a competent method for manufacturing metal parts of moderate complexity [1]. The literature in recent years has been enriched with various WAAM systems and technologies, emphasizing its growing relevance and application.

Ma et al. [2] introduced an innovative hybrid system that merges both subtractive and additive manufacturing methods, aimed at manufacturing large-scale thin-walled aluminum structures. Williams et al. [3] developed a specialized WAAM system engineered to produce large aircraft components, including wing spars and external parts for landing gears. In the work conducted by Li et al.’s research team [4], a robotic WAAM strategy was applied for the creation of inclined, multi-layer, multi-bead steel components. Lately, the WAAM method has been recognized for its critical role in rectifying surface imperfections in metallic parts.

According to the power source and wire-feeding mode, WAAM can be classified as gas tungsten arc additive manufacturing (GTA-AM) and gas metal arc additive manufacturing (GMA-AM). The WAAM process is celebrated for its cost efficiency, impeccable deposition density, superior characteristics, and enhanced productivity [3,5]. Cold Metal Transfer (CMT) technology stands out for its precise control over the heat input, as highlighted in earlier analyses [6]. Therefore, this study opted for CMT as the primary heat source in the WAAM process. Scholars such as Cong [7] and Gu [8,9] have effectively leveraged the CMT-WAAM approach for fabricating aluminum alloy components, addressing issues of porosity by either choosing the appropriate CMT variant or integrating interpass cold-rolling methods. Mehnen et al. [10] have extensively explored shape and manufacturing strategies in the CMT-WAAM domain, leading to the production of parts with complex surface finishes. Another experiment was designed and conducted by Remacheet al. [11] that mainly focused on exploring the sensitivity of material fatigue life to surface roughness and ultimate tensile strength (UTS)during the Laser Metal Deposition (LMD) process. Their findings consistently demonstrated that higher surface roughness led to a shorter fatigue life, while higher UTS values resulted in a longer fatigue life. In addition, Ding et al. [12] utilized the AM forming method to rapidly generate the basic configurations of 316 L stainless steel components and employed SM milling to achieve their machined surfaces, and their results indicated that the rapid cooling rate in the direct energy deposition process contributed to grain refinement and the precipitation of ferrite, which resulted in improvements in the hardness and tensile strength.

Challenges such as residual stresses and distortions significantly affect wire-feed AM processes due to the high levels of energy input, rapid deposition rates, and intense temperature gradients typical of these methods. While the CMT approach is favored in additive manufacturing for its minimal heat input, the back-and-forth deposition pattern tends to encourage the formation of columnar grains, adversely affecting the mechanical integrity of the fabricated items. Therefore, integrating Cold Metal Transfer with a pulse arc (CMT+P) in the additive manufacturing process targets the enhancement of microstructural properties and the improvement of mechanical properties.

In the field of AM, the appearance of deposited beads is crucial for assessing the quality of the final product. Therefore, it is essential to study the formation characteristics of these beads when subjected to various processing parameters in both CMT-WAAM and CMT+P-WAAM methods. This research focuses on analyzing how different deposition currents (ranging from 100 A to 175 A) affect the formation characteristics of beads and suggests an optimal range of parameters for achieving high-quality beads. Additionally, the effect of the CMT pulse arc on microstructure evolution is explored and analyzed. The paper is structured as follows: Section 2 introduces the experimentation overview; Section 3 details the experimental findings and discussions; Section 4 presents the effects of CMT+P and CMT arcs on the microstructure evolution; and the main conclusions are outlined in Section 5.

## 2. Experimentation Overview

### 2.1. Experimental System and Material

**Figure 1:**
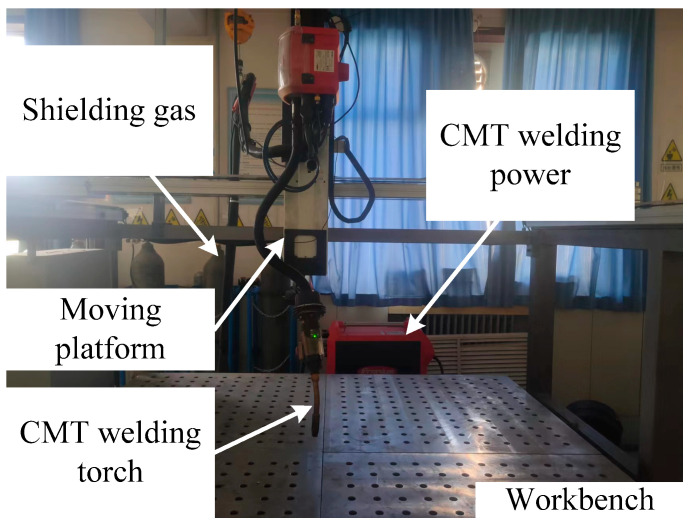
Schematic diagram of the CMT-WAAM setup.

The forming system used was a wire and arc additive manufacturing system composed of a model robot and a Synergic 5000 CMT (pulse) welding machine equipped with a wire-feeding system and welding torch, as illustrated in Figure 1. The welding material chosen was 1.2 mm diameter 316 stainless steel wire, and its chemical composition is displayed in Table 1. The base material was 316 stainless steel plates measuring 100 mm × 50 mm × 5 mm. Prior to welding, the plates were cleaned of oil, water, and other contaminants through mechanical grinding and then chemically cleaned. The removal of the oxide layer was achieved with an 8–10% NaOH solution, followed by neutralization with a 30% HNO_3_ solution after rinsing with water. Throughout the deposition process, the welding torch was maintained perpendicular to the substrate, with Ar being utilized as the shielding gas.

### 2.2. Experimental Procedure

This study primarily analyzed the effects of two key parameters on the formation characteristics of deposition beads in CMT-WAAM: the deposition current and the travel speed, as these are the principal variables in the CMT-WAAM process. Other parameters were kept constant for simplicity. The specific values and ranges of these parameters under analysis are presented in Table 2.

To establish the process window of the CMT-WAAM methodology, single-bead deposition experiments were designed and executed. Considering the relationship between the welding current and travel speed, the maximum deposition current and ideal forming characteristics of deposited beads were assessed. This assessment prioritized both their visual appearance and cross-sectional profiles. Specific settings of the experiments were as follows: the applied deposition current started from 100 A and increased in increments of 25 A until the maximum travel speed reached its threshold (at which point the deposition beads exhibited discontinuity). These experiments were conducted utilizing both CMT and CMT+P modalities for WAAM. To appraise the effect of the pulse arc on microstructure evolution during the CMT-WAAM process, the EBSD method was utilized. Samples were prepared utilizing electropolishing with a 10% vol HClO_4_ alcohol solution. Electropolishing parameters included a voltage of 30 V, a current of 1 A, and an electrolysis time of 15 s. EBSD images of the polished samples were obtained at 200× magnification with a 1 mm step size.

## 3. Results and Discussion

### 3.1. The Feasible Travel Speed Intervals for Each Deposition Current in the CMT-WAAM Process

**Figure 2:**
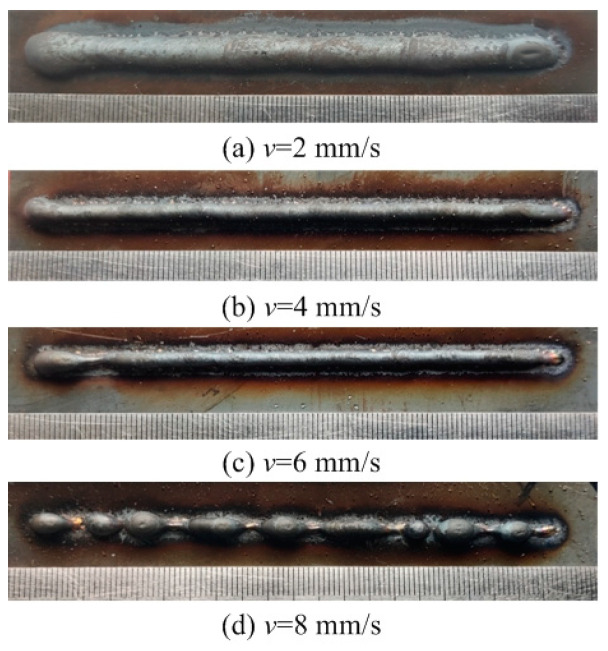
Surface appearance *I* = 100 A.

**Figure 3:**
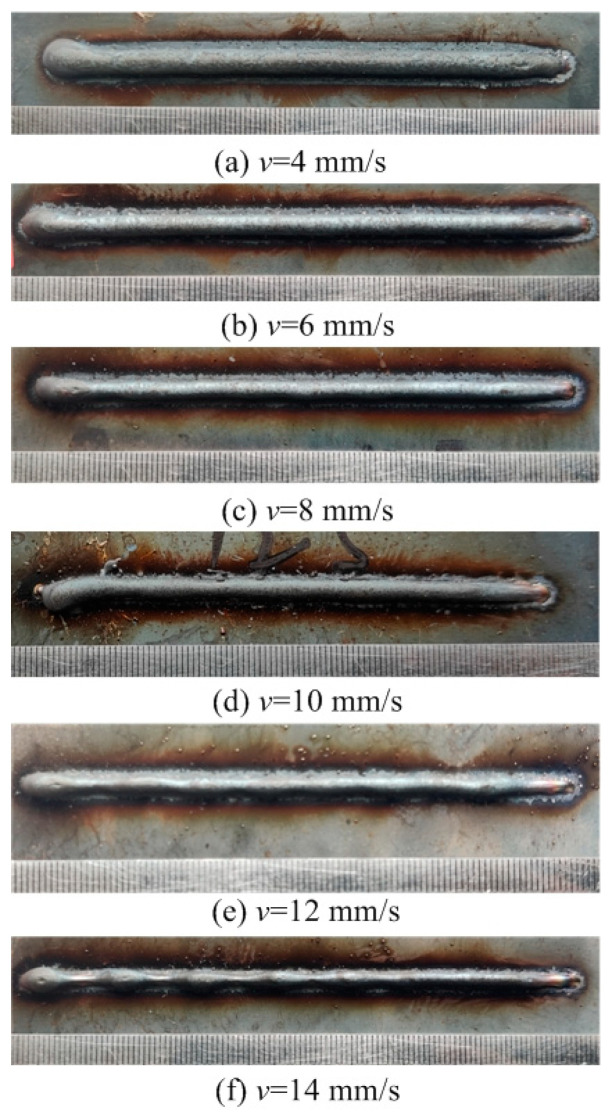
Surface appearance *I* = 125 A.

**Figure 4:**
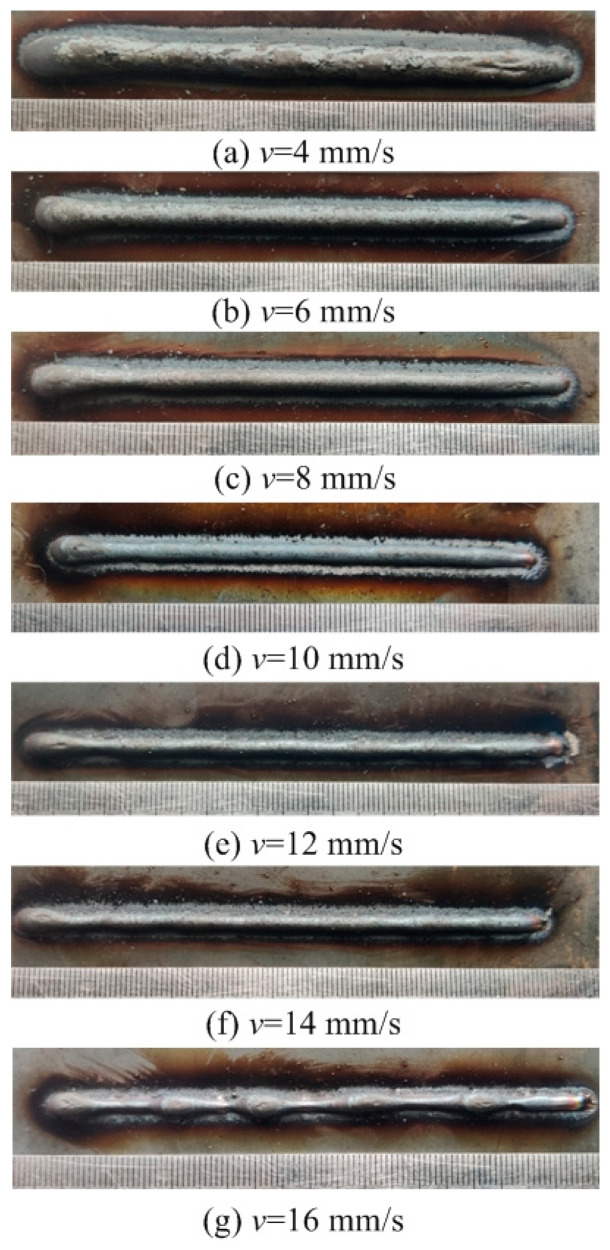
Surface appearance *I* = 150 A.

**Figure 5:**
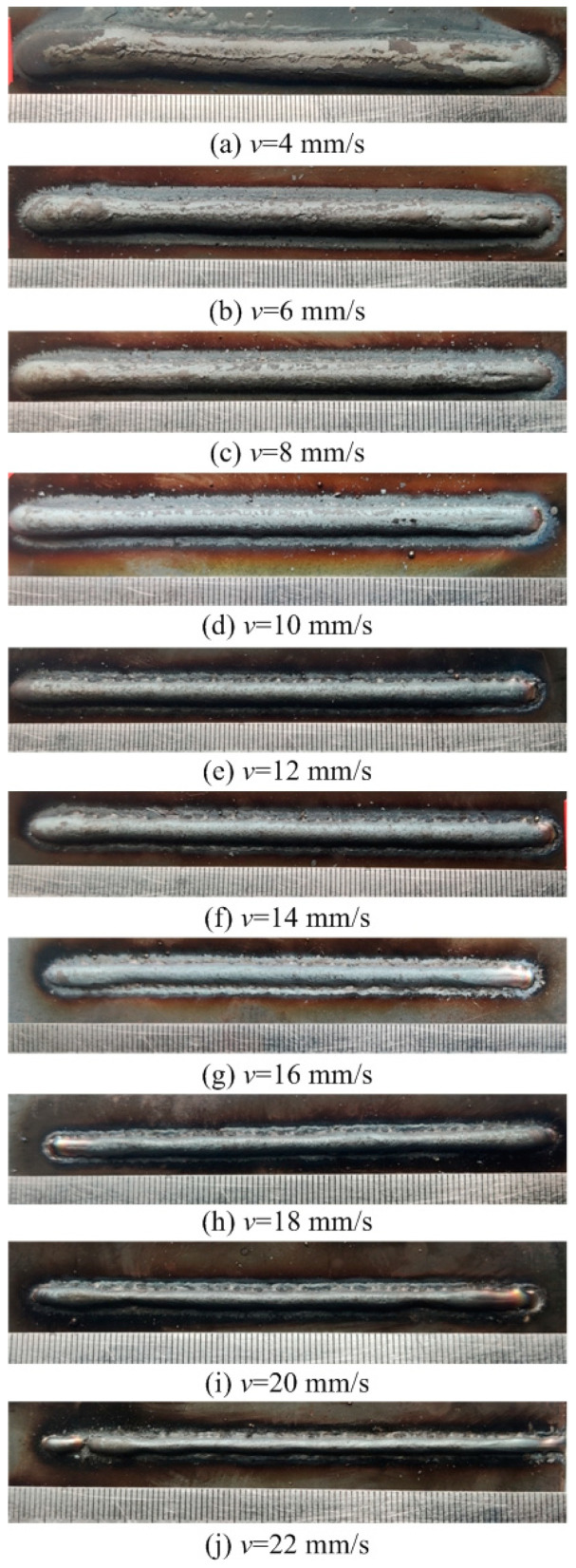
Surface appearance *I* = 175 A.

This section evaluates both the qualitative and quantitative findings of the single-layer samples created utilizing the CMT arc process, as illustrated in Figure 2, Figure 3, Figure 4 and Figure 5, viewed perpendicularly to the upper surface.

Observations across most samples indicate a minor presence of oxidation both on the surface of the deposition beads and in the heat-affected zone adjacent to the substrate. The oxide layer formed mostly after the specimens were removed from the print bed and allowed to cool to room temperature through natural convection without the presence of shielding gas or any other form of oxidation. This phenomenon was visually apparent as a gray hue on several samples (e.g., Figure 2c, Figure 3c–e, Figure 4c–f and Figure 5c–h). Notably, the oxidation layer’s coloration evolved as its thickness increased (e.g., Figure 2a, Figure 3a,b, Figure 4a,b and Figure 5a,b).

Four samples (Figure 2d, Figure 3f, Figure 4g and Figure 5j) exhibited a discontinuous weld bead formation. In instances such as the cases displayed in Figure 2d and Figure 3f, the droplets remained distinctly separate. Due to the significant height variations and discontinuities, these beads were considered unsuitable for AM applications and thus were excluded from subsequent analyses.

The primary metric for evaluation was the roughness of each bead along its length. For each sample, roughness measurements were conducted at two points and averaged to derive a final assessment value. A smoother bead surface, indicative of minimal roughness, is preferred to ensure consistent working distance and geometric precision during the layer-upon-layer deposition process.

**Figure 6:**
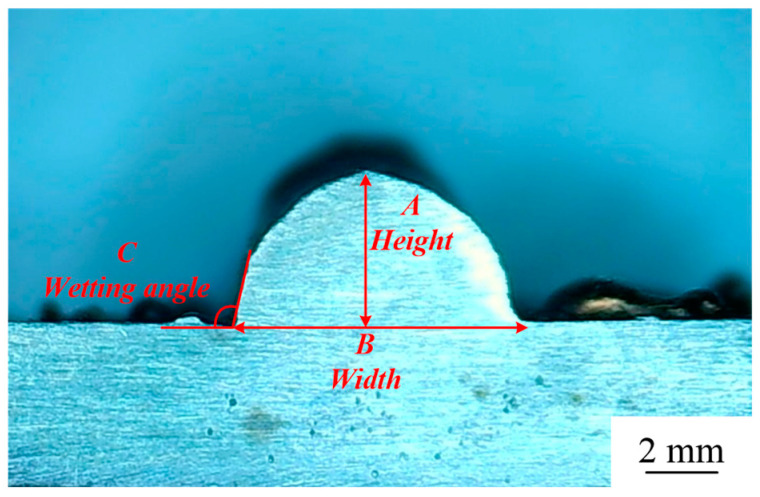
Sample geometry measurements in OM: (A) height, (B) width, and (C) wetting angle.

The samples were evaluated based on multiple criteria to identify trends and effects. The assessments focused on weld quality, characterized by bead roughness, width, and height. A bead characterized by a low surface roughness, devoid of significant height variations or waviness, facilitates the uniform deposition of successive layers, crucial for maintaining consistent working distances and geometric fidelity. The dimensions of bead width and height were measured to measure the effects of various processing parameters on the bead’s geometry. These measurements were performed utilizing Image J, an open-source image processing software, and the results are detailed in Figure 6.

**Figure 7:**
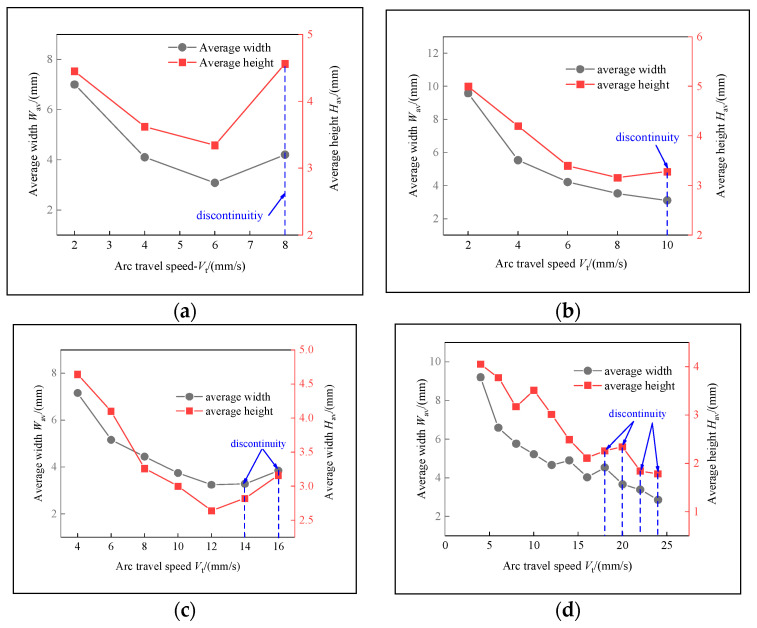
Feasible arc travel speeds under different deposition currents: (**a**) *I* = 100 A, (**b**) *I* = 125 A, (**c**) *I* = 150 A, (**d**) *I* = 175 A.

The range of viable wire feed speeds across the varying welding currents depicted in Figure 7 illustrates that as the welding current increased, the rate of metal deposition per unit of time also rose. Therefore, the permissible spectrum of arc travel speed expanded, moving from 6 mm/s at a welding current of 100 A to 16 mm/s at a welding current of 175 A.

Exploring the effect of the arc travel speed on the characteristics of deposition beads indicated significant findings, as presented in Table 3. These characteristics exhibited a well-defined, narrow, and intermittent nature, varying with each deposition current. The analysis indicated that both the width and height of the deposition beads decreased as the arc travel speed increased. This correlation was due to the prolonged duration of arc activity per unit length, elevating both the heat input and the volume of the melted wire. However, when the peak of the deposition bead nears the tip of the metal wire, any disruption in the deposition process may lead to an upward surge of the molten pool, potentially causing a short circuit in the CMT power supply. This scenario can result in the deposition process becoming unstable. Therefore, our study established a maximum deposition bead height of 8.5 mm, defining the minimum allowable arc travel speed. Conversely, excessively high arc travel speeds can result in an insufficient heat input for complete melting, producing a deposition bead characterized by a sequence of droplets, hence an intermittent bead performance. This condition sets the maximum allowable arc travel speed. The surface appearances of the discontinuous deposition beads as represented in Figure 2d, Figure 3f, Figure 4g and Figure 5j.

**Figure 8:**
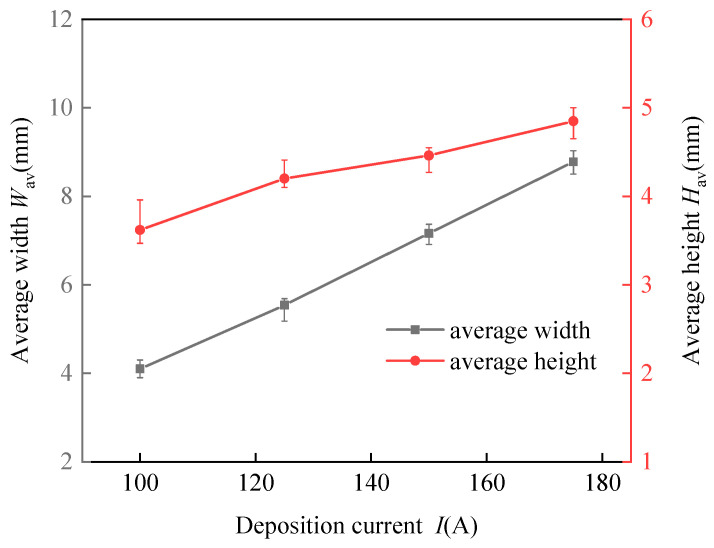
Effects of the deposition current on the width and height of the deposition beads at an arc travel speed of 4 mm/s.

The influences of different deposition currents on the deposition forming characteristics at a welding speed of 4 mm/s are depicted in Figure 8. As the deposition current increased, both the width and height of the deposition bead exhibited a growth trend. This observation suggests that an increase in the wire feed speed introduces more wire into the molten pool per unit time, expanding the pool’s volume. Despite this enlargement, the deposition bead’s width remained relatively unchanged throughout the process. This stability resulted in the deposition beads assuming a more significant and fuller shape due to surface tension forces. Nonetheless, when the wetting angle exceeded 90 degrees, the ability of the beads to spread out slightly deteriorated. In such instances, the contour of the well-defined bead does not blend smoothly with the substrate line, posing challenges for depositing multi-layer single-pass walls.

As the arc travel feed speed continued to increase, changes in the force condition of the molten pool occurred and the effect of gravity appeared to be more significant, comparable to surface tension. The width of the molten pool remained constant, leading to the molten metal sinking and finally extending along the width direction. This action resulted in the wetting angle surpassing 90 degrees, which is less than ideal for creating multi-layer single-pass walls.

### 3.2. The Feasible Travel Speed Intervals for Each Deposition Current in the CMT+P-WAAM Process

During the additive manufacturing process, the role of the heat input cannot be overstated regarding its impact on the microstructure and mechanical properties. Leveraging CMT with a pulsed arc for deposition is crucial for achieving the most optimally enhanced mechanical properties and refined microstructures.

**Figure 9:**
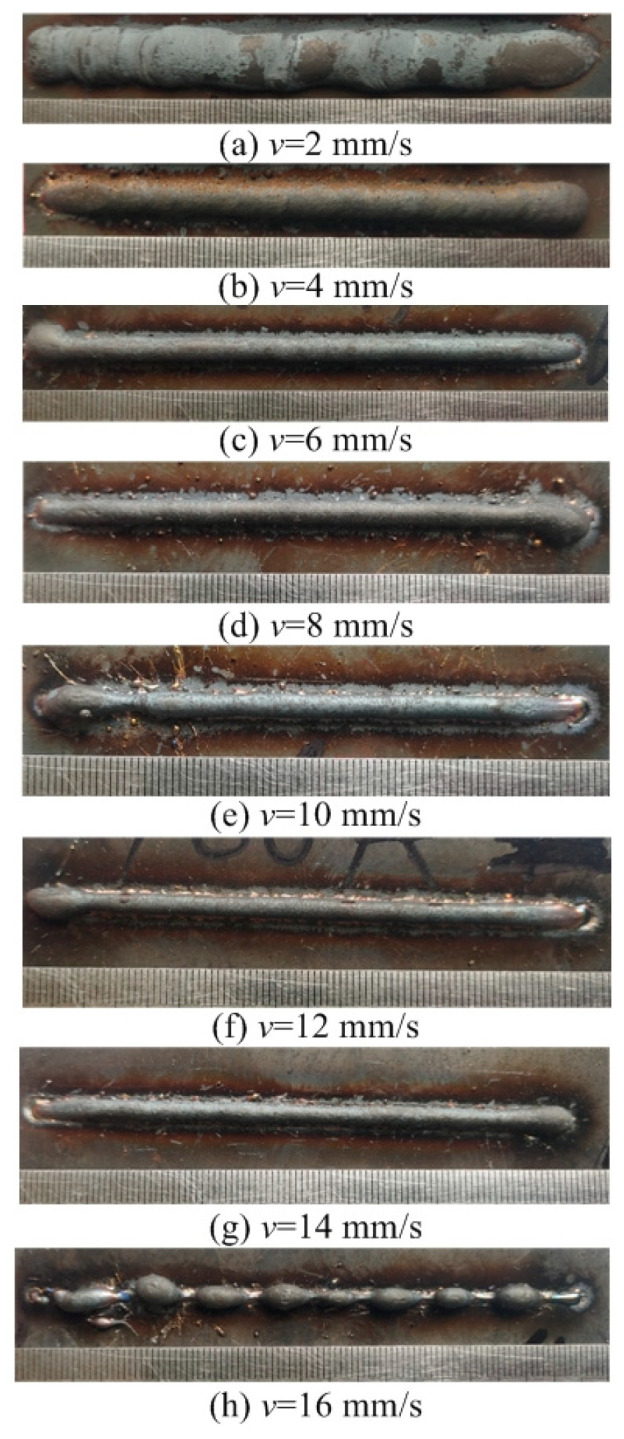
Surface appearance *I*_p_ = 100 A.

**Figure 10:**
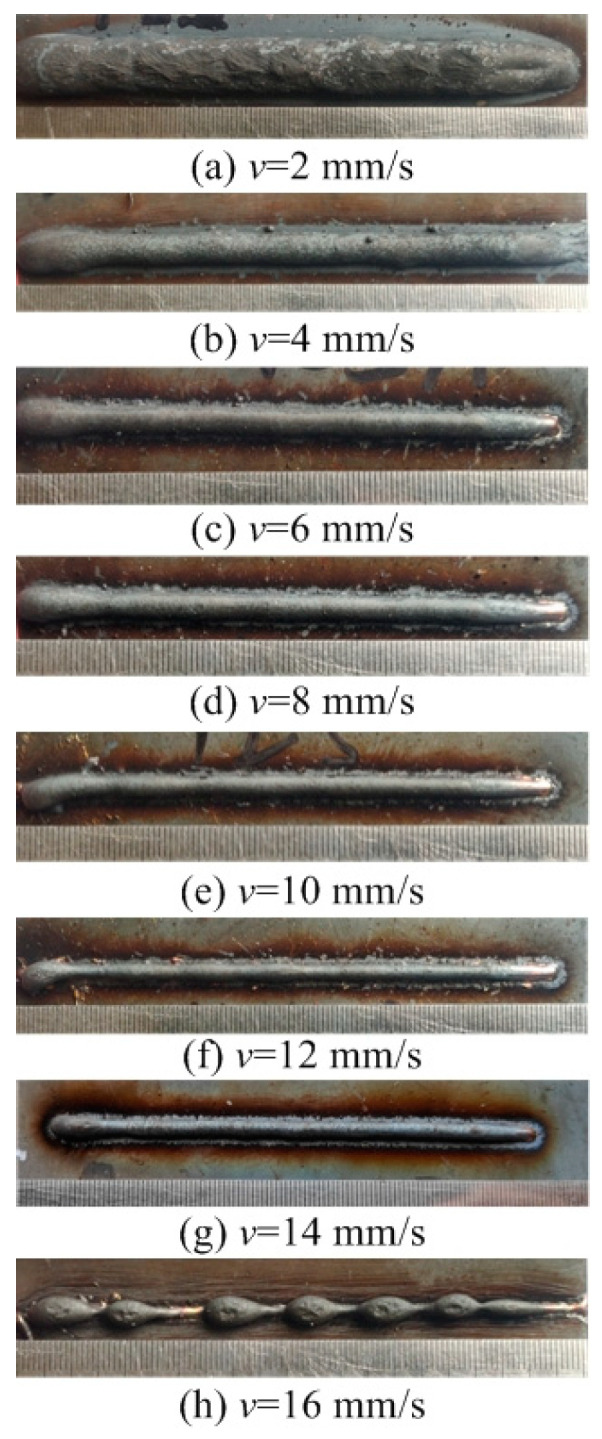
Surface appearance *I*_p_ = 125 A.

**Figure 11:**
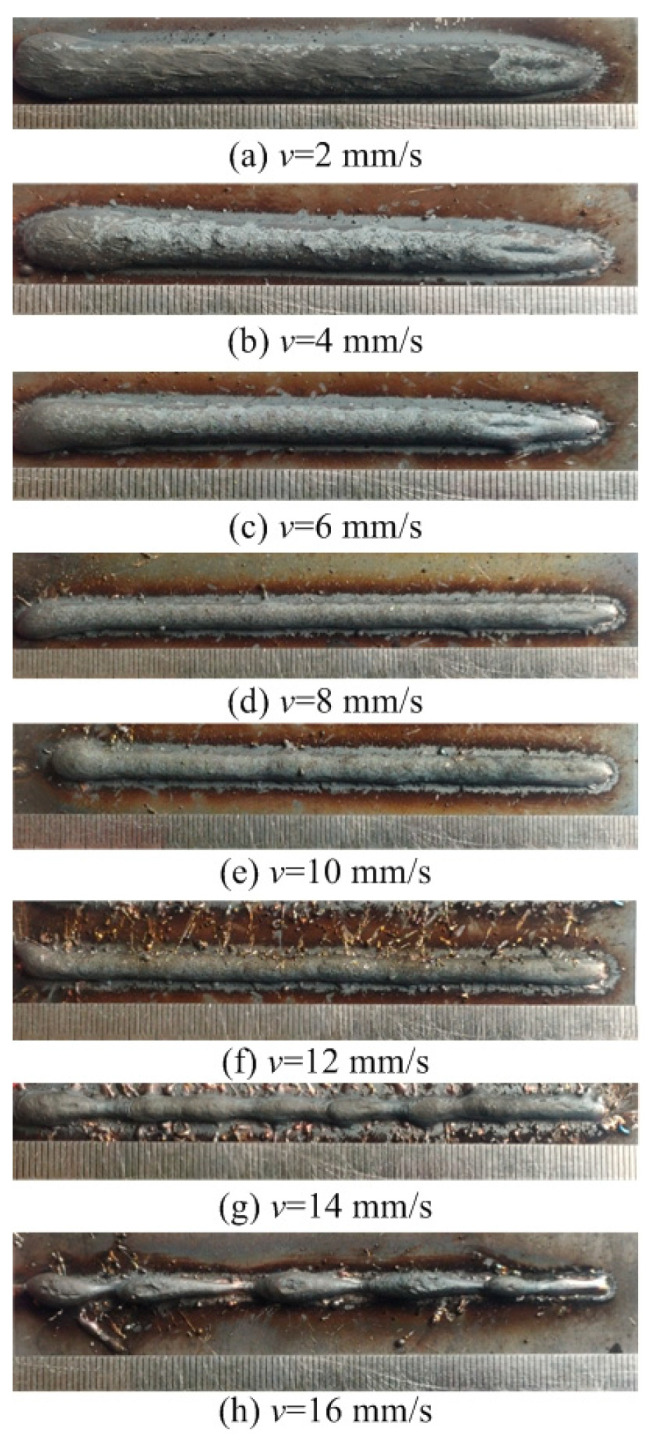
Surface appearance *I*_p_ = 150 A.

**Figure 12:**
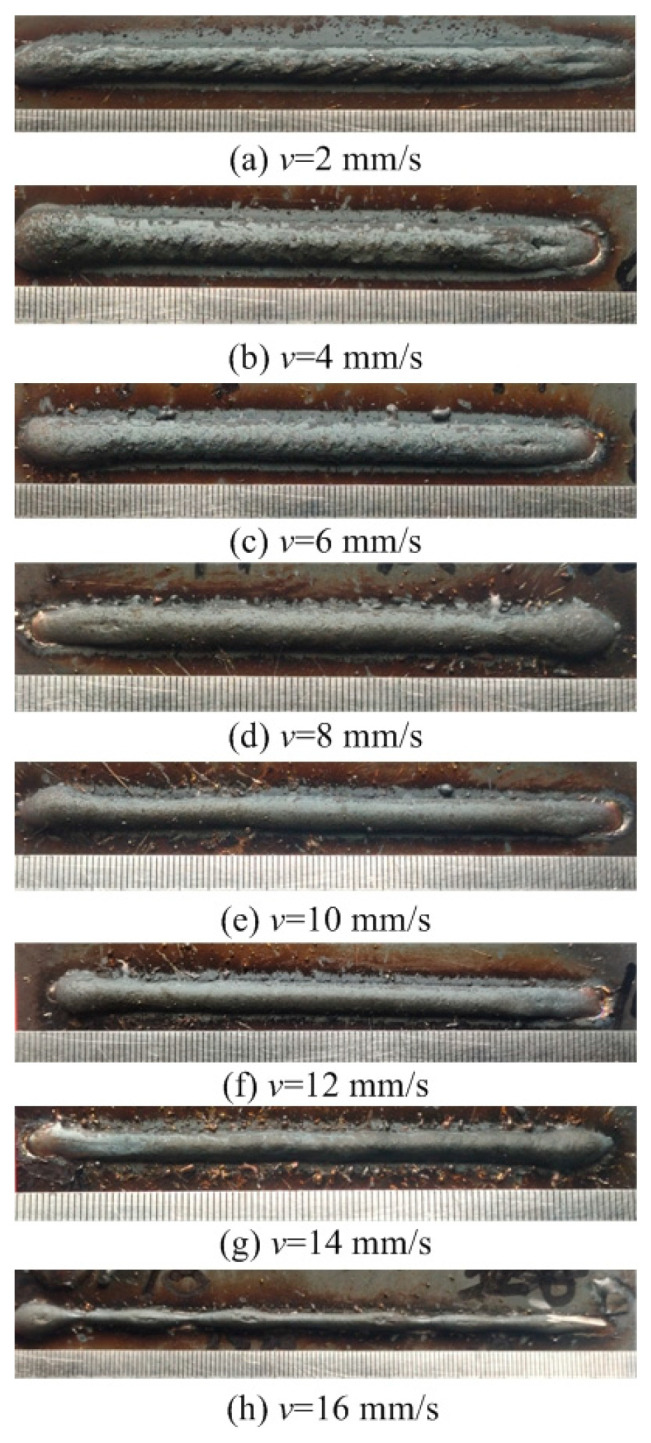
Surface appearance *I*_p_ = 175 A.

To ensure that the experiments were comparable, the average value of the pulsed current (*I*_p_) was made equal to the current value of the direct-current CMT arc. This equivalence was determined through the following formula [13]:

$$I_{p}=\frac{I_{m}\times t_{m}+I_{j}\times t_{j}}{t_{m}+t_{j}}$$

where $I_{m}$ is the peak current, $I_{j}$ is the base current, $t_{m}$ is the time duration of the peak current, and $t_{j}$ is the time duration of the base current. The CMT pulse arc process parameters are listed in Table 4. The other factors were fixed as constants. The effects of varying average pulsed current values on the morphology of the deposited layers are depicted in Figure 9, Figure 10, Figure 11 and Figure 12. Observations from these figures indicate that utilizing a CMT pulsed arc significantly broadens the acceptable range of travel speeds, resulting in deposited layers that are more robust and have superior surface profiles.

**Figure 13:**
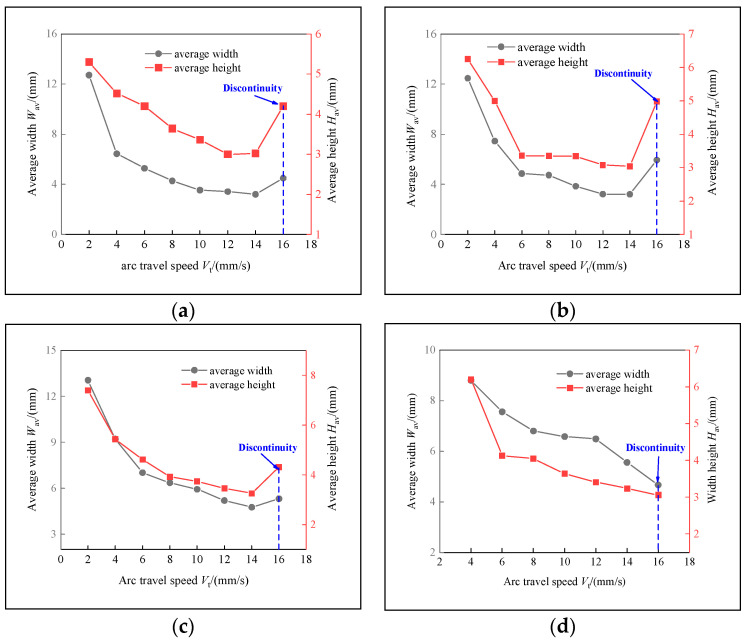
Feasible arc travel speeds under different deposition currents: (**a**) *I_p_* = 100 A, (**b**) *I_p_* = 125 A, (**c**) *I_p_* = 150 A, (**d**) *I_p_* = 175 A.

In Figure 13, it is demonstrated that employing a CMT pulse arc in wire and arc additive manufacturing notably enhances the process window. This means that under the same conditions, the arc travel speed can effectively range from 2 to 14 mm/s, with an optimal manufacturing speed of 4–10 mm/s. This range is particularly chosen because at lower speeds, the deposited layer tends to be wider and taller, which compromises the quality of the formation and introduces a risk of instability in the CMT arc and wire additive manufacturing process. On the other hand, at higher speeds, the process may result in a series of molten droplets, leading to discontinuous deposition layers, as depicted in Figure 9h, Figure 10h, Figure 11h and Figure 12h, that challenge the subsequent deposition layer.

**Figure 14:**
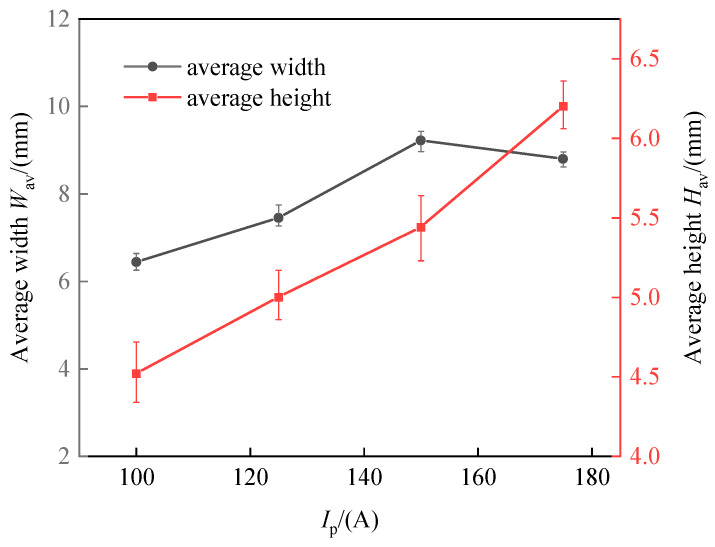
Effects of the deposition current of the CMT pulse arc on the width and height of the deposition beads.

Figure 14 indicates the effect of the average pulse current on the dimensions of the deposited beads. Figure 14 demonstrates that with a steady arc travel speed, an increase in the deposition current results in a significant increase in both the width and height of the beads. This observation aligns with the behaviors noted during the CMT arc and wire additive manufacturing process.

In summary, the CMT pulse arc and wire additive manufacturing approach does not significantly affect the formation characteristics of the deposited beads. The CMT pulse arc, through its precise control over the heat input, is particularly advantageous for constructing single-pass multi-layer thick walls. In addition, the CMT pulse arc’s introduction promotes grain refinement due to its precise heat management, a topic that will be elaborated upon in Section 4. Table 5 lists the present work and some other recent studies on traditional WAAM technology using similar filling materials.

## 4. Microstructure Characterization

**Figure 15:**
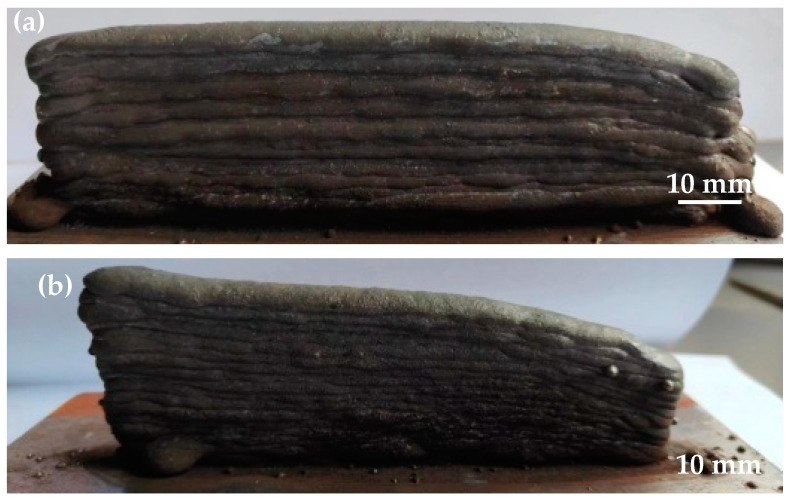
Macroscopic morphology of the as-deposited wall: (**a**) CMT, (**b**) CMT+P.

The overall macroscopic appearance of the wall structure immediately after deposition, as illustrated in Figure 15b, demonstrated a remarkably clean and uniform weld bead without any evidence of metal flow or structural collapse on the surface, highlighting the effective compatibility of the CMT+P method with 316 L stainless steel in the WAAM context. Notably, the average structure height was approximately 20 mm, presenting a difference to the results reported in [19], where a 50-layer deposition process utilizing 316 L wire of a 0.8 mm diameter through laser cladding achieved a height of approximately 60 mm. This comparison suggests that a relatively enhanced deposition efficiency was achieved in our experiment compared to the reference mentioned above.

**Figure 16:**
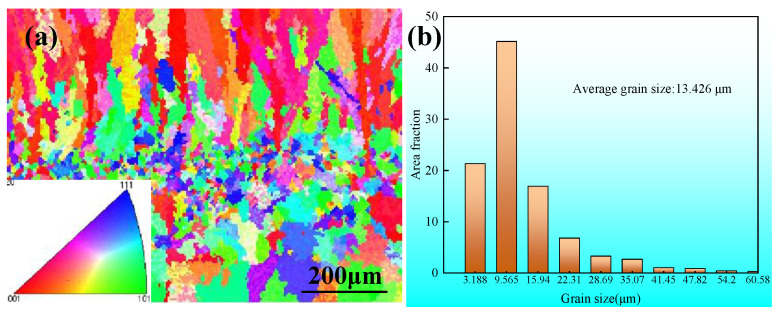
(**a**) Inverse pole figure and (**b**) grain size data for the middle region of the CMT-WAAM samples.

**Figure 17:**
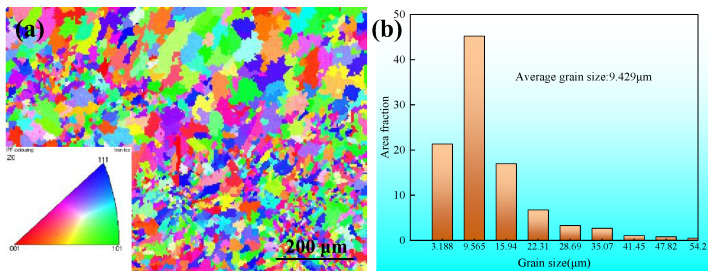
(**a**) Inverse pole figure and (**b**) grain size data for the middle region of the CMT+P-WAAM samples.

The granular size and directional orientation are critical in enhancing the mechanical integrity of additive-manufactured components [20,21]. To further study the microstructure of the stainless steel fabricated through both CMT and CMT+P-WAAM methods, EBSD analyses were conducted, as depicted in Figure 16 and Figure 17. Predominantly, the deposited wall exhibited a characteristic layer-belt microstructure, as illustrated in Figure 16. This structural formation is attributed to the sequential layering and solidification of liquid metal, with each new addition being deposited atop the previously solidified layer. The specified height of each layer closely matched the vertical displacement of the welding apparatus following each pass. The heat from transition droplets, as well as the arc heat, re-melted a portion of a deposited layer and promoted the formation of a molten pool on top of the deposited layer. This layer served as a foundational platform for nucleation, leading to the occurrence of heterogeneous nucleation atop the layer. Concurrently, the arc force propelled the transient droplets into the molten pool at high velocities, creating a circular stirring motion. This action disrupted the formation of larger dendrites, resulting in the emergence of finer dendrites.

The inverse pole figure (IPF) illustrated in Figure 16a demonstrates a sample fabricated utilizing the CMT arc and wire additive manufacturing method. This sample exhibited an evident preferred orientation along a specific direction, attributed to the highest temperature gradient during the process. Previous studies, as mentioned in the literature [22], have confirmed that the direction of construction aligns with this maximum temperature gradient. Conversely, Figure 17a indicates that samples produced utilizing the CMT+P method were characterized by a mix of fine and coarse grains, with grain orientations appearing randomly and lacking any significantly preferred direction. The adoption of the CMT+P pulse arc in creating components through CMT-WAAM has been demonstrated to enhance the grain orientation and refine grain size [22].

Regarding the grain size in the components created through CMT-WAAM, the average grain size was found to be approximately 13.426 μm, as depicted in Figure 16b, with the largest grain size being over 60.58 μm. However, when employing the CMT+P arc for component fabrication, both the average and the maximum grain sizes were reduced to approximately 9.429 μm and over 54.2 μm, respectively. This reduction indicates that the use of CMT+P-WAAM leads to a refinement in the grain size of the components.

## 5. Conclusions

In this work, the effect of CMT and CMT+P on the formation characteristics and microstructure refinement of 316 austenitic stainless steel during CMT-based WAAM was analyzed, and our results can be summarized in several key points as follows:

(1) The application of both direct current and pulse current proved effective in manufacturing stainless steel components and enhancing their microstructure through CMT-based WAAM.
(2) The analysis of specimens from multiple layers indicated no visible discontinuity between the layers, indicating thorough fusion across layers.
(3) The dimensions of the deposition beads, i.e., both their width and height, were observed to decrease as the travel speed increased. Experimental determination identified the optimal range of travel speeds for each deposition current, alongside the maximum allowable wire feed speed. Notably, a travel speed of up to 16 mm/s at a deposition current of 175 A was achievable when utilizing CMT pulse arcs.
(4) When the CMT mode arc was used, the average grain size was 13.426 μm, but when the pulse arc was 9.429 μm, the grain size decreased obviously.

## Figures and Tables

**Table 1:** Chemical composition of the 316 stainless steel metal wire (wt %).

Elements	C	Cr	Ni	Mo	Si	Mn	P	Cu	Fe
(wt.%)	0.0014	18.74	11.82	2.67	0.56	1.55	0.03	0.17	Bal.

**Table 2:** Basic deposition process parameters.

Process Parameters	Value
Deposition current *I*/A	100, 125, 150, 175
Arc length *L*/mm	10
Flow rate of the shielding gas *Q* (L/min)	15
Diameter of the metal wire *d*/mm	1.2
Arc travel speed *v* (mm/s)	2

**Table 3:** Forming characteristics of the deposition beads with each deposition current and arc travel speed.

Exp. (No.)	Deposition Current *I* (A)	Arc Travel Speed *V*_t_ (mm/s)	Forming Characteristic
Figure 2a	100	2	Well formed
Figure 2b	100	4	Well formed
Figure 2c	100	6	Narrow width
Figure 2d	100	8	Discontinuous
Figure 3a	125	2	Well formed
Figure 3b	125	4	Well formed
Figure 3c	125	6	Well formed
Figure 3d	125	8	Well formed
Figure 3e	125	10	Narrow width
Figure 3f	125	12	Discontinuous
Figure 4a	150	2	Well formed
Figure 4b	150	4	Well formed
Figure 4c	150	6	Well formed
Figure 4d	150	8	Narrow width
Figure 4e	150	10	Narrow width
Figure 4f	150	12	Narrow width
Figure 4g	150	14	Discontinuous
Figure 5a	175	2	Well formed
Figure 5b	175	4	Well formed
Figure 5c	175	6	Well formed
Figure 5d	175	8	Well formed
Figure 5e	175	10	Well formed
Figure 5f	175	12	Well formed
Figure 5g	175	14	Well formed
Figure 5h	175	16	Narrow width
Figure 5i	175	18	Narrow width
Figure 5j	175	20	Narrow width

**Table 4:** Basic deposition process parameters for the CMT pulse arc.

CMT Pulse Arc Parameters	Value
Peak current *I*_m_/A	200
Base current *I*_j_/A	100
Duty cycle *K*/%	50%
Pulse *f*/Hz	20

**Table 5:** Comparison of the present work with other typical studies on the deposition rate.

Authors	Method	Filling Material	Wire Feed Speed
Colegrove et al. [14]	GMA-AM	0.8 mm diameter wire of ER70S-6	10 m/min
Xiong et al. [15]	GMA-AM	1.2 mm diameter wire of H08Mn2Si	6 m/min
Yilmaz and Ugla [16]	GTA-AM	0.8 mm diameter wire of ER308LSi	3.5 m/min
Paskual et al. [17]	GTA-AM	1.2 mm diameter wire of AISI 316 L	Not mentioned
Han et al. [18]	GTA-AM	1.2 mm diameter wire of H08Mn2Si	5 m/min
This study	CMT-AM	1.2 mm diameter wire of 316	Not mentioned

## Data Availability

All data generated or analyzed during this study are included in this published article. The experimental data in this article can be used for scientific research, teaching, etc.
